# A silent gigantic solitary fibrous tumor of the pleura: case report

**DOI:** 10.1186/1749-8090-6-122

**Published:** 2011-09-29

**Authors:** Nobuyuki Furukawa, Bert Hansky, Jost Niedermeyer, Jan Gummert, Andre Renner

**Affiliations:** 1 Department of Cardiothoracic Surgery, Heart and Diabetes Center North Rhine-Westphalia, Georgstr. 11, 32545 Bad Oeynhausen, Germany; 2 Department of Pulmonology, Krankenhaus Bad Oeynhausen, Wielandstr. 28, 32545 Bad Oeynhausen, Germany

## Abstract

Solitary fibrous tumor of the pleura is a rare mesenchymal tumor, representing less than 5% of all neoplasms associated with the pleura. A 57-year-old man had general malaise without chest symptoms for 1 month. A chest roentgenogram and computed tomography showed a giant mass in the left thorax. Although the tumor compressed the descending aorta and other mediastinal structures strongly, thereby shifting them to the right side, the patient had no symptoms except malaise. The tumor was successfully resected via two separate thoracotomies. The tumor was measured (20 cm × 19 cm × 15 cm) and weighed (2150 g). The tumor was histologically and immunohistochemically diagnosed as benign. Although SFT is benign, a long follow-up period is essential as even patients with complete resection are at risk of recurrence many years after surgery.

## Background

Solitary fibrous tumors (SFT) of the pleura are rare intrathoracic neoplasm. Immunohistochemical analysis has confirmed that SFTs originate from mesenchyme underlying the mesothelial layer of the pleura. Although they are usually asymptomatic, larger tumors occupying a large space in the thoracic cavity, present more commonly with symptoms such as dyspnea, chest pain and malaise. Although the tumor was large enough to push the descending aorta and other mediastinal structures to the right, our patient displayed no symptoms other than malaise. We successfully resected the huge tumor via two separate thoracotomies. One year later, the patient is in good health without tumor recurrence.

## Case presentation

**Figure 1:**
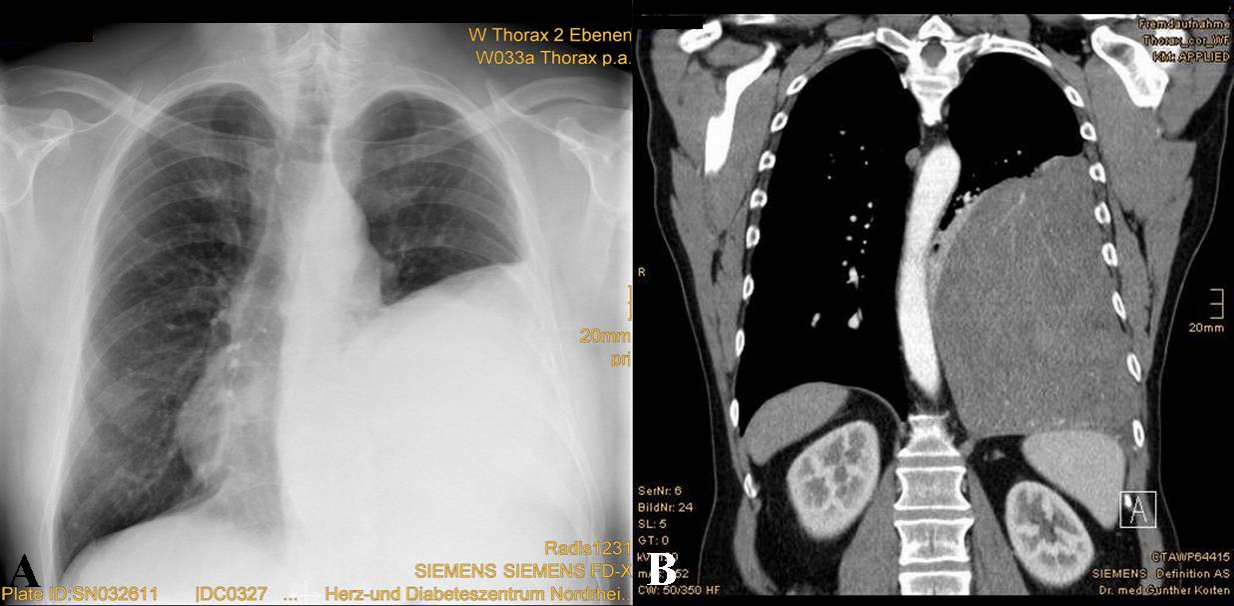
**Chest radiography and CT scan images**. (A) Initial chest radiography revealed a large well-circumscribed mass in the left thorax. (B) Initial contrast-enhanced computed tomography showed a huge homogeneous, sharply defined mass compressing the aorta.

A 57-year-old man was referred to a hospital because of progressive general malaise for a month. His medical history was unremarkable and he had no history of exposure to asbestos. At physical examination, breath sounds were absent on the left lower region. A roentgenogram showed a giant tumor in the left thorax (Figure 1A). The heart appeared to be compressed towards the right side. He had no other chest complaints, such as cough, chest pain, and dyspnea. Computed tomography (CT) revealed a well-circumscribed homogeneous mass, which compressed the descending aorta (Figure 1B). The hematological and biochemical findings were normal. Bronchofiberoscopy showed stenosis of the left lower lobar bronchus from extraluminal compression. Bronchoscopic cytology revealed no abnormal findings and no evidence of bronchitis. CT-guided biopsy demonstrated fibrotic soft tissue without evidence of malignancy but the appearance of the specimen did not have enough diagnostic strength. Spirometry showed the following results: vital capacity, 2.4 L (49% of predicted); forced expiratory volume in a second, 1.7 L (42% of predicted). Results of blood gas analysis were also within normal limits. The patient was referred to our institution for surgical treatment of a suspected SFT.

**Figure 2:**
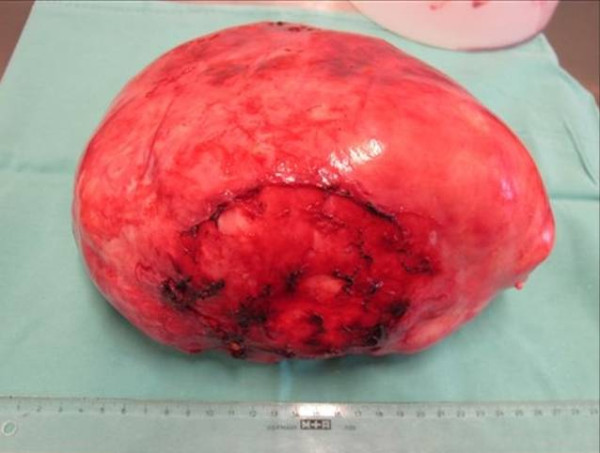
**The gigantic encapsulated solitary fibrous tumor of the pleura, weighed 2150 g and measured 20 cm × 19 cm × 15 cm**.

**Figure 3:**
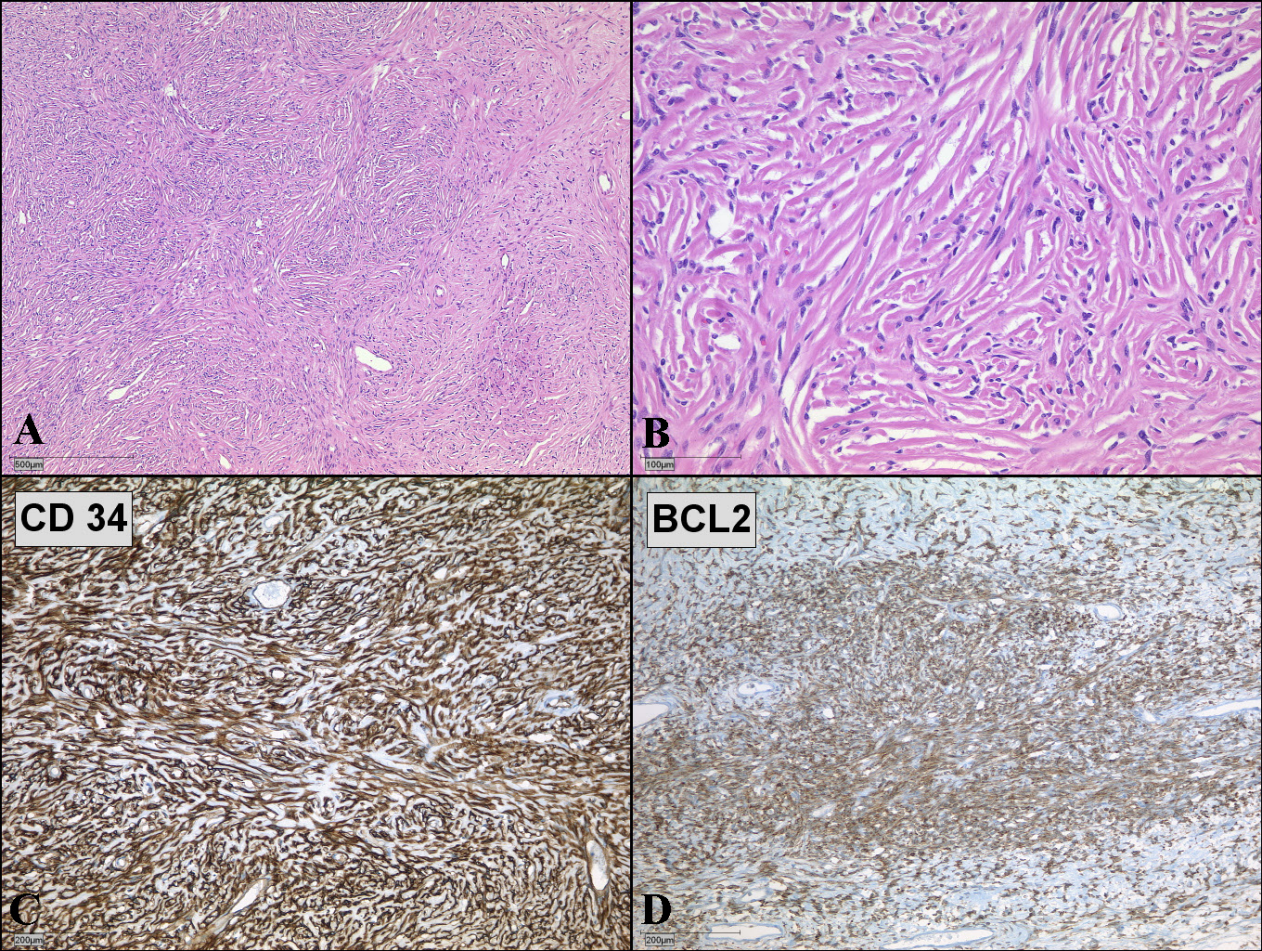
**Microscopic examination of solitary fibrous tumor of the pleura**. (A, B) Microscopic specimen of the tumor shows solid proliferation of spindle-shaped fibroblastic cells in a patternless pattern. (Hematoxylin and eosin; magnification 40× and 200×) (C, D) Spindle-shaped tumor cells show strong positivities for immunohistochemical staining with CD34 (C) and BCL2 (D).

Left posterolateral thoracotomy through the fifth and eighth intercostal spaces was performed for the resection of the tumor. We choose the fifth intercostal space as our initial Thoracotomy site. Upon entering the pleura we could easily visualize the encapsulated circumscribed gigantic tumor. The tumor was large (20 cm × 19 cm × 15 cm), extended from the thoracic aperture to the diaphragm, and caused atelectasis of the lower lobe of the left lung. An additional incision through the eighth intercostal space was made to dissect the tumor away from the diaphragm. Because the tumor had strongly attached to the lingula of the left lung, atypical wedge resection of the lingula was performed. The main vascular pedicle of the tumor was identified in the hilum of the lung. There were several small feeder vessels from the diaphragm. The tumor was fixed to the diaphragm, and we dissected it precisely either by ligation or occlusion with diathermy. The main pedicle from the hilum was ligated with nonabsorbable ties. The tumor weighed 2150 g, and appeared smooth surfaced and well-circumscribed on macroscopic examination (Figure 2). Histologically, the tumor appeared to be composed of a varying proportion of spindle-shaped cells and collagen. The neoplastic cells displayed vesicular nuclei with demarcated nuclear membranes, and dispersed chromatin. Mitoses were rare, and immunoreactivity to vimentin, CD34, and Bcl2 were positive; cytokeratin was negative (Figure 3). The tumor was pathologically diagnosed as benign localized fibrous tumor of the pleura. The left lung expanded completely and pulmonary function recovered to the normal level after removal of the giant tumor. The postoperative course was uneventful and the patient was discharged 12 days after the operation.

## Discussion

Although diffuse pleural tumors or mesothelioma are common, solitary fibrous pleural tumors are rare. SFT represents less than 5% of pleural tumors [1] and occurs most often in the visceral (80%) and parietal pleura (20%) [2]. It has been recently considered to originate from the mesenchymal cells of the submesothelial connective tissue of the pleura. According to immunohistochemical analysis, SFT of the pleura is positive for vimentin, CD34, CD99, and Bcl2, which are markers of mesenchymal cells; but it is negative for cytokeratin, which is found in mesotheliomas. These results indicate that SFT originates from mesenchymal cells rather than mesothelial cells [1]. England et al. listed classical criteria of malignant SFT, which is also useful for diagnosis, as follows: more than 4 mitotic activity in 10 high-powered fields, necrosis, high cellularity, and pleomorphism [3].

The common presentations are relatively small tumors less than 10 cm in diameter in an asymptomatic patient, discovered incidentally on chest roentgenograms. For tumors larger than 10 cm, occupying a large space and compressing other thoracic structures may cause symptoms such as dyspnea, chest pain, cough, and fatigue. Uncommonly hypertrophic pulmonary osteoarthropathy and hypoglycemia are also caused. Hypertrophic osteoarthropathy, called Pierre Marie-Bamberger syndrome, is associated with the abnormal production of hyaluronic acid by the tumors. Hypoglycemia is caused by the insulin-like growth factor 2, which is secreted by the tumors [2].

In our case, the gigantic tumor weighed 2150 g. Large tumors, heavier than 2 kg, have been rarely reported [4,5]. Larger tumors are more likely to be malignant and are associated with the worst prognosis [3,5,6]. The presence of symptoms and pleural effusion, which are also reported as factors associated with malignancy, are more likely in patients with large tumors [3,7,8]. This indicates that the prognosis depends on the complete resectability of the tumor and on the diagnosis of malignancy.

Occasional recurrences have been reported not only in malignant cases but also in benign cases, even though it is small percentage (1.4%) [7]. In our case, postoperative adjuvant chemotherapy was not performed, because histologically the tumor was identified as a benign SFT, and surgical margins revealed no residual tumor. The role of adjuvant chemotherapy in SFTs remains uncertain. Although complete resection was achieved, close follow-up is indicated because of the possibility of recurrence.

## Conclusion

We report a case of a patient with a gigantic solitary fibrous tumor (SFT) of the pleura. Although the tumor compressed the lung, the descending aorta and other mediastinal structures strongly, the patient had no symptoms except malaise and had normally worked as a furniture remover. We successfully resected the huge solitary fibrous tumor of the pleura via two separate thoracotomies. Although SFT is benign, a long follow-up period is essential as even patients with complete resection are at risk of recurrence many years after surgery.

## Consent

Written informed consent was obtained from the patient for publication of this case report and any accompanying images. A copy of the written consent is available for review by the Editor-in-Chief of this journal.

## Abbreviations

SFT: solitary fibrous tumor; CT: computed tomography.

## Competing interests

The authors declare that they have no competing interests.

## Authors' contributions

NF carried out the manuscript and collected references. JN and JG helped to revise the manuscript. BH and AR underwent the operation. All Authors read and approved the final manuscript.
